# Poor child complementary Feeding Practices in northwest Ethiopia: Finding from the Baseline Survey of Nutrition Project, 2016

**DOI:** 10.1186/s13052-019-0747-2

**Published:** 2019-12-02

**Authors:** Zegeye Abebe, Amare Tariku, Gashaw Andargie Bikes, Molla Mesele Wassie, Kedir Abdela Gonete, Tadesse Awoke, Ejigu Gebeye, Azeb Atnafu Gete, Melkie Edris Yesuf, Yigzaw Kebede, Kassahun Alemu, Abebaw Addis, Esmael Ali Muhammad, Solomon Mekonnen Abebe, Aysheshim Kasahun belew, Melkamu Tamir Hunegnaw, Melkitu Fentie, Adane Kebede, Kindie Fentahun Muchie

**Affiliations:** 1 Department of Human Nutrition, Institute of Public Health, College of Medicine and Health Sciences, University of Gondar, Gondar, Ethiopia; 2 Department of Health Services Management and Health Economics, Institute of Public Health, College of Medicine and Health Sciences, University of Gondar, Gondar, Ethiopia; 3 Department of Epidemiology and Biostatistics, Institute of Public Health, College of Medicine and Health Sciences, University of Gondar, Gondar, Ethiopia; 4 Department of Reproductive Health, Institute of Public Health, College of Medicine and Health Sciences, University of Gondar, Gondar, Ethiopia

**Keywords:** Infants and young children, Dietary diversity, Dabat HDSS site, Ethiopia

## Abstract

**Background:**

Improving infant and young child feeding practices is critical to improved nutrition, health, and development of children. Ethiopia adopted the WHO recommendations of child feeding practices and developed the national guideline. In spite of this fact, only few children start and received appropriate complementary feeding based on the recommendation. Therefore, the study aimed to determine dietary diversity score and its associated factors among under five children at Dabat Health and Demographic Surveillance System site (HDSS), northwest Ethiopia.

**Methods:**

A cross-sectional community based study was carried out from February to June 2016. All children aged 6–59 months old who lived in HDSS site were included in the survey. Odds ratio (OR) with the corresponding 95% confidence interval (CI) was calculated to show the strength of association. Finally, variables with a *P-*value of < 0.05 were considered statistically significant..

**Results:**

In this study, a total of 3433 children were included. About 34.87% (95%CI: 33.27, 36.49%) of the children received adequately diversified diet. The odds of receiving adequately diversified diet was higher among children whose mother had secondary and above education (AOR = 6.51; 95%CI: 4.95, 8.56), had antenatal care (AOR = 1.90; 95%CI: 1.60, 2.26) and postnatal care visits (AOR = 1.31; 95%CI: 1.00, 1, 72), and children who feed with their family (AOR = 1.39; 95%CI: 1.17, 1.65). However, a lower dietary diversity score was observed among younger children; 6–11 months old (AOR = 0.59; 95%CI: 0.41, 0.85), and children from food insecure household (AOR = 0.76; 95%CI: 0.63, 0.92).

**Conclusions:**

Diversified diet feeding practice is low in Dabat HDSS site. Age of the child, maternal education, antenatal and postnatal care visits, and household food insecurity were significantly associated with dietary diversity of children. Hence, ensuring household food security and enhancing the coverage of maternal health care utilization are recommended to increase dietary diversity of children.

## Background

Malnutrition, in its various form, remains a pressing and significant health problem of children in Ethiopia (1). It affects mortality and ill-health along the entire continuum of care from early childhood to adulthood (2). It is thus clear that the prevention of young child undernutrition is a long-term investment that will benefit the current generation as well as their children (3). The first two years of life is called a critical period to ensure the child’s development through optimum feeding practices (4, 5). If children are undernourished before they reach the age of 2 years, they could suffer from irreversible physical and mental damage and this will undoubtedly influence their future health and wellness. Thus, improving infant and young child feeding (IYCF) practices is therefore critical to improved nutrition, health, and development of children (2, 6).

It is confirmed that exclusive breast-feeding adequately provides for children’s energy and nutrient needs in the first six months of life. However breast-milk alone cannot meet the increased energy and nutrient requirements as children get older (7). Thus, the World Health Organization (WHO) recommends that infants should be exclusively breastfed for the first 6 months of life, after which they are introduced to appropriate complementary foods as they continue to breastfeed (6). Complementary food introduction should be timely, nutritionally adequate, appropriate, and safe for the development of children’s full human potential. Hence, complimentary food should be made from variety of foods groups to ensure daily nutritional requirements of children (8).

Dietary diversity is a potentially useful indicator for nutrient adequacy of a diet and nutritional status (9–11), nevertheless the coverage of diversified diet in infants and young children continues as a challenge for developing countries. For instance, 26.5 and 41% of children in Nigeria and Kenya received adequately diversified diet, respectively (12) (13). Despite child undernutrition is pervasive in Ethiopia, inappropriate complementary feeding remained a deep rooted malpractice. According to the Ethiopia Demography and Health Survey (EDHS) 2016 report, 7% of the children received the minimum feeding standards, 14% received adequate dietary diversified diet and 45% the recommended meal frequency. Similarly, other local studies also noted that feeding of diversified diet is well under the recommendation, 25% in Gondar, 35% in Bihar Dar, and 45% in Addis Ababa (1).

To improve child feeding practices and nutritional status of children, the government of Ethiopia has been implemented different activities. Accordingly, the country adopted the WHO recommendations of child feeding practices and developed the national guideline of IYCF to improve child’s nutrition and health status (14). However, a few children start and received appropriate complementary feeding based on the recommendation. Therefore, investigating the extent of appropriate child feeding practice and associated factors are important to monitor the in place interventions thereby to make an evidence-based decision. - Also, there is need to update the existing literatures using recent and representative surveillance data.

## Methods

### Study setting

A community based cross-sectional study was conducted from February to June 2016 in Dabat HDSS site. The HDSS site is located in Dabat district, northwest Ethiopia. The district has an estimated population size of 185, 3076, and has 6 health centers, 31 rural and 5 urban health posts. The HDSS site has been running since 1996, and hosted by the University of Gondar. The surveillance site covers thirteen randomly selected kebeles (four urban and nine rural kebeles, the smallest administrative area in Ethiopia) in different ecological zones (high land, middle land, and low land**).**

### **Study participants,** data collection tool and procedures

All children aged 6–59 months old who lived in HDSS site were included in the survey. The data were collected from mothers of children using a structured questionnaire via interview. Questioners were adopted from previous studies with some modification to fit the local context. The questionnaire consistent of varied nutritional, dietary intake, health care utilization, and morbidity characteristics. The questionnaire was first developed in English and translated into Amharic then back translated into English to maintain consistency. Pretest was done on 5 % participants out of the study area. Experienced 38 data collectors and seven field supervisors who have been permanently working HDSS site were involved in the data collection process. Tree days training on interviewing technique and data collection process was given to data collectors and supervisors. During data collection intensive supervision was carried out by investigators and supervisors.

### Variable measurements

Child dietary diversity score (DDS) was assessed based on seven food groups recommended by the WHO IYCF guideline. Food groups included in the measurement were grains/roots/tubers; legumes and nuts; dairy products; flesh foods (meats/fish/poultry); eggs; vitamin A-rich fruits and vegetables (VAFV); and other fruits and vegetables (OFV). The DDS was constructed by assigning one point to each of the defined food groups, for a maximum of seven points. Then, we defined adequate dietary diversity as consumption of food from at least four different food groups (DDS ≥ 4).

Household food security status (HFSS) was assessed by using the standardized questionnaire developed by Food and Nutritional Technical Assistance (FANTA). A month recall period with two types of questions, nine occurrence questions followed by three frequency of occurrence questions for each event, was used to determine the status. Finally, households were categorized as food secured when the score was ≤1, and food insecure for a score ≥ 2.

### Data processing and analysis

The collected data were checked and entered into Epi info version 7 and exported to STATA version 14 statistical software for analysis. Descriptive statistics were carried out and the result was presented using text and Tables. A binary logistic regression model was fitted to identify factors associated with adequate dietary diversity. Variables with a *p*-value of less than 0.2 in the bivariable analysis were fitted into the multivariable logistic regression analysis. Both crude odds ratio (COR) and adjusted odds ratio (AOR) with the corresponding 95% confidence interval (CI) were calculated to show the strength of association. Finally, a *P-*value of < 0.05 was used to determine presence of statistically significant association.

## Results

### Characteristics of the parents and their children

In this study, a total of 3433 children were included. Almost half, (49.2%), of the children were male and one fourth were in the age range of 36–47 months. Regarding maternal characteristics, 70.3% were unable to read, while 86.13 and 85.49% were housewives and married, respectively (Table 1).

**Table 1:** Characteristics of the parents and their children at Dabat HDSS site, northwest Ethiopia, February to June 2016 (*n* = 3433)

Variables	Frequency	Percentage
**Sex of the child**		
Male	1689	49.20
Female	1744	50.80
**Child age (in months)**		
6–11	371	10.81
12–23	802	23.36
24–35	774	22.55
36–47	839	24.44
48–59	647	18.85
**ANC visit**		
No	1202	35.01
Yes	2231	64.99
**Maternal education**		
Unable to read and write	2404	70.03
Primary education	681	19.84
Secondary and above	348	10.14
**Mother’s occupation**		
Farmer	624	18.18
Housewife	2333	86.13
Merchant	286	8.33
Others	190	5.53
**Marital status**		
Single	323	9.41
Married	2935	85.49
Other^*^	175	5.10
**Residence**		
Urban	588	17.13
Rural	2845	82.87
PNC visit		
No	3153	91.84
Yes	280	8.16

ANC = Antenatal care, PNC = Postnatal care*widowed, separated

### Child feeding practices

In this study, about 34.87% (95%CI: 33.27, 36.49%) of the children received adequately diversified diet. Of all children, 5.62% started complementary feeding early before six months and 59.63% started complementary feeding at six months. About 18.64% of the children received meat and flesh foods 24 h before the date of survey. Similarly, consumption of foods rich in vitamin A or iron remains low among children in the study area. About 27.06% of children consumed foods rich in vitamin A, and 18.64% consumed iron rich foods 24 h before the date of interview. However, about 98.8% of the children predominantly consumed cereals over a 24-h period. Finally, among 6–11 age of children, 62% of them received the recommended meal frequency (Table 2). Regarding child nutritional status, around 7.9% of the children were wasted and 37.4% were stunted.

**Table 2:** Feeding practices and family food choice for their children at Dabat HDSS site, northwest Ethiopia, February to June 2016 (*n* = 3433)

Variables	Frequency	Percentage
**Breast feeding**		
No	1640	47.77
Yes	1793	52.23
**Time of complementary introduction**		
Before six months	193	5.62
At six months	2047	59.63
After six months	1193	34.76
**Preparation of Complementary Feeding**		
For the child only	832	24.24
With the family	2601	75.76
**Dietary Diversity**		
Adequate	1197	34.87
Inadequate	2236	65.13
**Child feeding style**		
With the family	1395	40.64
Separately	2038	59.36
**Food source**		
Home garden	2483	73.83
Market	949	79.95
**Frequency of food buying (*n*** **= 1180)**		
Daily	20	1.69
2–3 times weekly	95	8.05
Weekly	269	22.80
Once in two weeks	180	15.25
Monthly	616	52.21
We didn’t buy	20	1.69
**Reasons not to buy food (n = 11)**		
Too costly	4	36.36
The money sent for other purpose	3	27.27
The food is unavailable	2	18.18
Others	2	18.18
**Distance between home and the market (*n*** **= 1183)**		
< 1 km	589	49.79
1-4 km	216	18.26
5-10 km	27	2.28
11-20 km	95	8.03
21-30 km	89	7.52
31-40 km	114	9.64
41-50 km	30	2.54
> 50 km	23	1.96
**Transportation to market**		
On foot	1173	99.66
Others (car and animals)	4	0. 34
**Food preference/ Food choice to their children**		
No	2794	81.40
Yes	639	18.60
**Types of Food preference to their children (*n*** = 639)*		
Fruit and vegetables	325	50.8
Meat and fish	447	69.95
Egg, milk and milk product	476	74.49
Honey	262	41.00
Packed foods	306	47.89
Cereals and food make from it	582	91.08

*Multiple choice

### Factors associated with DDS among children

Table 3 shows factors associated with adequate dietary diversity of children aged 6–59 months. Accordingly, age of the children, maternal education, maternal ANC and PNC follow up, household food security status and method of child feeding were associated with DDS.

**Table 3:** Factors associated with DDS among children at Dabat HDSS site, northwest Ethiopia, February to June 2016 (*n* = 3433)

Variables	DDS		COR 95%CI	AOR 95%CI
	Adequate	Inadequate		
**Age of children (in months)**				
6–11	113	258	0.73 (0.56, 0.96)	0.59 (0.41, 0.85)^*^
12–23	270	532	0.85 (0.68, 1.05)	0.78 (0.58, 1.06)
24–35	270	504	0.89 (0.72, 1.11)	0.80 (0.61, 1.04)
36–47	302	537	0.94 (0.76, 1.16)	0.90 (0.71, 1.13)
48–59	242	405	1.00	1.00
**Sex of the children**				
Male	610	1079	1.00	1.00
Female	587	1157	0.89 (0.78, 1.03)	0.92 (0.79, 1.07)
**Mother’s education**				
Unable to read and write	635	1769	1.00	1.00
Primary education	304	377	2.25 (1.88, 2.68)	2.11 (1.76, 2.53)^*^
Secondary and above	258	90	7.98 (6.18, 10.32)	6.51 (4.95, 8.56)^*^
**ANC visit**				
No	278	924	1.00	1.00
Yes	919	1312	2.33 (1.99, 2.73)	1.90 (1.60, 2.26)^*^
**PNC visit**				
No	1067	2086	1.00	1.00
Yes	130	150	1.69 (1.32, 2.16)	1.31 (1.00, 1.72)^*^
**Methods of child feeding**				
With family	526	869	1.23 (1.07, 1.42)	1.39 (1.17, 1.65)^*^
The child alone	671	1367	1.00	1.00
**Methods of CF preparation**				
Separately	323	509	1.00	1.00
With the family	874	1727	0.80 (0.69, 0.94)	1.00 (0.81, 1.23)
**Food security**				
Secured	994	1714	1.00	1.00
Unsecured	203	522	0.67 (0.56, 0.80)	0.76 (0.63, 0.92)^*^
**Food preference**				
No	954	1840	1.00	1.00
Yes	243	396	1.18 (0.99, 1.41)	1.12 (0.92, 1.36)

ANC = Antenatal care, PNC = Postnatal care

*indicate significant at *p*- value less than 0.05 in multivariable logistic analysis

The odds of receiving adequately diversified diet among younger children (6–11 months) were 41% (AOR = 0.59; 95%CI: 0.41, 0.85) lesser compared to the older children (48–59 months). Mothers who had secondary and above educational status were 6 times (AOR = 6.51; 95%CI: 4.95, 8.56) more likely to provide adequately diversified diet to their children compared to mothers who had no education. Similarly, mothers who attended primary school were about 2 times (AOR = 2.11; 95%CI: 1.76, 2.53) more likely to practice adequate diversified diet than those who had no formal education. The likelihood of diversified diet was higher among children whose mothers who had ANC (AOR = 1.90; 95%CI: 1.60, 2.26) and PNC follow ups (AOR = 1.31; 95%CI: 1.00, 1,72).

Finally, those children from food insecure household were 24% less likely to received adequately diversified diet compared to children from food secured household (AOR = 0.76; 95%CI: 0.63, 0.92).

## Discussion

Diversified diet is presumed to provide all the essential nutrients to children which in turn has paramount importance to attain their normal growth and development (15). Nevertheless, only 34.87% of the children in Dabat HDSS site had diversified diet. This indicates that two-third of children with undiversified diet are at risk of poor micronutrient intake and growth failure. This result in poor developmental, cognitive and behavioral outcomes. It is evident that children with low DDS were more likely to be stunted (12).

Despite the fact that the findings from this study is higher than a report from Southern (18.8%) (8) and Benishangul Gumuz (23.7%) (16) regions of Ethiopia, all findings are much lower than the WHO recommendation, greater than 80% (17). This indicates various and rigorous actions needed to scale up for appropriate child feeding practices. Because complementary foods should be timely, adequate, safe and appropriate to tackle the most pressing nutritional problem of the children (18).

Mothers who had ANC and PNC follow up were more likely to provide diversified diet to their children. This finding is supported with other study in Ethiopia (19, 20). ANC and PNC follow up may supported with complementary feeding education, and this may encourage mothers to retain the existing beneficial food habits and add other foods which may help to meet their children nutritional needs, healthy feeding practices, offering of energy dense and adequate amount of complementary foods (21, 22). In addition, successful complementary feeding education reinforces the existing cultural pattern and brings about qualitative improvement by using available food resources (23).

A maternal education level, primary education and above was significantly associated with dietary diversity score of children; educated mothers more likely to provide adequately dietary diversified diet to their children compared to those mothers who were unable to read and write. It is clear that education is the primary intervention in all aspects of health promotion and prevention of health related problems. Educated mothers are more likely to use health care facilities and can easily understand the nutrient requirement of their children and they can also plan, select and prepared energy dense and adequate complementary foods to their children. Specifically, maternal education is the corner stone of appropriate child feeding practices and prevention of hygiene related diseases (2).

The age of the child is also found to have an association with dietary diversity score. Younger children were less likely to receive the recommended diversified diet compared to the older one. This finding is supported by that of another study conducted in Ethiopia (20, 24). This is probably because younger infants’ are mostly breastfed, so the need for a frequent feeding of extra solid food is not perceived as important or a priority by mothers and caretakers for feeding infants of this age. In addition, older children have the chance of eating and adapting family diet as age advance.

The likelihood of having adequate dietary diversity was higher among children who feed with their family compared to children feed alone. This is because family characteristics such as parental role modeling for eating, parental encouragement, parents’ food preferences, regular meal patterns and feeding practices encourage a higher intake of diversified diet among children (25, 26).

Finally, household food insecurity is associated with dietary diversity score of children. This finding is supported with other studies (13, 27). This is due to the availability and utilization of diversified foods depends upon the economic contexts of the household and affordability of such foods. Thus, food inscure households may affect time of introduction of complementary feeding and adherence to the IYCF recommendation. Therefore, low dietary diversity may be due to limited income available to purchase foods, and reducing the variety of foods consumed and preparing cereal based monotones diet are the coping strategies adopted in the face of food insecurity (28).

The study attempted to show child feeding practices in a well-defined population representing rural northwest Ethiopia. However, the study has some limitations. First, the study did not consider the quantity of food consumed by the children and a single 24-h recall did not indicate the usual dietary habit of the children. Second, even though adequate training was given data collectors and supervisors, there might still be a social desirability and recall bias in reporting the type of food given to children.

## Conclusions

Diversified diet feeding practice is low in the HDSS site. Consumption of meat, fish and foods rich in vitamin A and iron remains low among children in the study area. However, Cereals were the predominant food groups consumed over a 24-h period. Age of the child, maternal education, maternal ANC and PNC follow up visits, household food security status and method of child feeding is significantly associated with DDS of the children. Hence, various actions need to scale up the current practices of child feeding by ensuring household food security and enhancing the coverage of maternal health care utilization are recommended to increase dietary diversity of children.

## Data Availability

Data will be available upon request from the corresponding author.

## Abbreviations

AOR: adjusted odd ratio
CI: confidence interval.
EDHS: Ethiopia Demography and Health Survey
FANT: Food and Nutrition Technical Assistance
HDSS: Health and Demographic Surveillance System
WHO: World Health Organization; BMI: body mass index

## References

[1] Central Statistical Agency [Ethiopia] and ICF International. Ethiopia Demographic and Health Survey 2011, Addis Ababa, Ethiopia and Calverton,Maryland, USA: Central Statistical Agency and ICF International. Available from https://dhsprogram.com/pubs/pdf/FR328/FR328.pdf: 2016.

[2] WHO. Infant and young child feeding: model chapter for textbooks for medical students and allied health professionals. Available from https://apps.who.int/iris/handle/10665/44117: 2009. 23905206

[3] World Bank (2006). Repositioning nutrition as central to development: a strategy for large scale action.

[4] Gewa CA, Leslie TF (2015). Distribution and determinants of young child feeding practices in the East African region: demographic health survey data analysis from 2008–2011. J Health Popul Nutr.

[5] Ndubuka J, Ndubuka N, Li Y, Marshall CM, Ehiri J. Knowledge, attitudes and practices regarding infant feeding among HIV-infected pregnant women in Gaborone, Botswana: a cross-sectional survey. BMJ Open. 2013. 10.1136/bmjopen-2013-003749 PMC3845062 24293206

[6] UNICEF (2012). Programming guide: infant and young child feeding.

[7] Vossenaar M, Solomons NW (2012). The concept of“critical nutrient density” in complementary feeding: the demands on the“family foods” for the nutrient adequacy of young Guatemalan children with continued breastfeeding. Am J Clin Nutr.

[8] Kassa T, Meshesha B, Haji Y, Ebrahim J (2016). Appropriate complementary feeding practices and associated factors among mothers of children age 6–23 months in southern Ethiopia, 2015. BMC Pediatr.

[9] Zhao W, Yu K, Tan S, Zheng Y, Zhao A, Wang P (2017). Dietary diversity scores: an indicator of micronutrient inadequacy instead of obesity for Chinese children. BMC Public Health.

[10] Ruel MT (2003). Is dietary diversity an indicator of food security or dietary quality? A review of measurement issues and research needs. Food Nutr Bull.

[11] Divya N, Rajanish KV, Malavika J, Sharma A (2018). The study of dietary diversity score in children between 6 months to 23 months: a hospital based study. Int J Contemp Pediatr.

[12] Ogechi UP, Chilezie OV (2017). Assessment of dietary diversity score, nutritional status and socio-demographic characteristics of Under-5 children in some rural areas of Imo state, Nigeria. Mal J Nutr.

[13] Macharia TN, Ochola S, Mutua MK, Kimani-Murage EW (2018). Association between household food security and infant feeding practices in urban informal settlements in Nairobi, Kenya. J Dev Orig Health Dis.

[14] Federal Ministry of Health FHDE (2004). National Strategy for infant and young child feeding.

[15] United Nations Children’s Fund, World Health Organization, The World Bank. UNICEFWHO-World Bank Joint Child Malnutrition Estimates. Geneva; The World Bank, Washington: UNICEF, New York; WHO, 2012.

[16] Ayana D, Tariku A, Feleke A, Woldie H (2017). Complementary feeding practices among children in Benishangul Gumuz region, Ethiopia. BMC Res Notes.

[17] World Health Organization. Infant and young child feeding: a tool for assessing national practices, policies and programmes. 2003.

[18] Abeshu MA, Lelisa A, Geleta B (2016). Complementary feeding: review of recommendations, feeding practices, and adequacy of homemade complementary food preparations in developing countries – lessons from Ethiopia. Front Nutr.

[19] Temesgen H, Negesse A, Woyraw W, Mekonnen N (2018). Dietary diversity feeding practice and its associated factors among children age 6–23 months in Ethiopia from 2011 up to 2018: a systematic review and meta-analysis. Ital J Pediatr.

[20] Dangura D, Gebremedhin S. Dietary diversity and associated factors among children 6–23 months of age in Gorche district, Southern Ethiopia: Cross-sectional study. BMC pediatrics. 2017;17(1):6. 10.1186/s12887-016-0764-x PMC5223415 28068965

[21] Imdad A, Yakoob MY, Bhutta ZA (2011). Impact of maternal education about complementary feeding and provision of complementary foods on child growth in developing countries. BMC Public Health.

[22] Lassi Zohra S, Das Jai K, Zahid Guleshehwar, Imdad Aamer, Bhutta Zulfiqar A (2013). Impact of education and provision of complementary feeding on growth and morbidity in children less than 2 years of age in developing countries: a systematic review. BMC Public Health.

[23] Yohannes B, Ejamo E, Thangavel T, Yohannis M. Timely initiation of complementary feeding to children aged 6–23 months in rural Soro district of Southwest Ethiopia: a cross-sectional study. BMC pediatrics. 2018;18(1):17. 10.1186/s12887-018-0989-y PMC5793361 29386008

[24] Belew AK, Ali BM, Abebe Z, Dachew BA. Dietary diversity and meal frequency among infant and young children: a community based study. Ital J Pediatr. 2017;43(1):73. 10.1186/s13052-017-0384-6 PMC5558775 28810887

[25] Hursti U-KK (1999). Factors influencing children's food choice. Ann Med.

[26] Roos E, Lehto R, Ray C (2012). Parental family food choice motives and children’s food intake. Food Qual Prefer.

[27] Saha KK, Frongillo EA, Alam DS, Arifeen SE, Persson LÅ, Rasmussen KM (2008). Household food security is associated with infant feeding practices in rural Bangladesh. J Nutr.

[28] Agbadi Pascal, Urke Helga Bjørnøy, Mittelmark Maurice B. (2017). Household food security and adequacy of child diet in the food insecure region north in Ghana. PLOS ONE.

